# Mapping the sequence preference of the generalist class II lanthipeptide synthetase ProcM by mRNA display

**DOI:** 10.64898/2026.08.19.745792

**Published:** 2026-10-18

**Authors:** Yao Ouyang, Hassan Nadeem, Yuki Goto, Diwakar Shukla, Wilfred A. van der Donk

## Abstract

The biosynthetic machineries of ribosomally synthesized and post-translationally modified peptides (RiPPs) are often substrate tolerant. A remarkable example is the class II lanthipeptide synthetase ProcM, which naturally functions as a generalist enzyme that has not evolved to use a specific substrate during its evolutionary history. Although ProcM has been studied extensively, the sequence features associated with productive modification remain underexplored. In this study, we use the ultrahigh-throughput mRNA display technique to map the sequence compatibility of ProcM across a focused library. This approach expands the landscape of ProcM reactivity beyond native substrates and individually characterized variants. Machine learning (ML) analysis revealed that the dataset contains sequence patterns associated with ProcM reactivity, with models achieving a balanced accuracy of 0.73. This moderate predictive performance contrasts with the near-perfect accuracy reported for specialized enzymes. This suggests that predicting substrate compatibility for generalist enzymes is more challenging due to uneven representation of reactive and nonreactive sequences and limitations of the available data. Our results provide a high-throughput view of ProcM reactivity and highlight differences with previous high-throughput studies on substrate selectivity of RiPP modification enzymes. Future studies will need to assess whether these differences are common when comparing generalist with specialist enzymes.

**Table of Contents Graphic:**

